# Circulating miR-326 and miR-532-3p: novel biomarkers for early gestational diabetes mellitus detection with high diagnostic accuracy

**DOI:** 10.1007/s40618-026-02829-z

**Published:** 2026-02-05

**Authors:** Hui-Zhen Lin, Zhi-Kun Liang, Jun-Ju Huang, Yong Yang, Yan Huang, Zhi-Shen Wu, Jin-Lin Tan, Sheng Huang, Shi-Han Ruan, Bing-Yi Xu, Ze-Fan Ruan, Tao-Sheng Huang, Qi-Guang Wang, Long Xie

**Affiliations:** 1 Department of Clinical Laboratory, The First Hospital of Putian, Fujian Medical University Clinical Teaching Hospital, 351100 Putian, China; 2 Research Institute, DAAN Gene Co., Ltd, No. 19 Xiangshan Road, 510665 Guangzhou, China; 3 The Medicine and Biological Engineering Technology Research Centre of the Ministry of Health, 510663 Guangzhou, China; 4 College of Life Science and Technology, Huazhong University of Science and Technology, 430074 Wuhan, China; 5 Department of Clinical Laboratory, Hunan Provincial People’s Hospital, The First Affiliated Hospital of Hunan Normal University, 410005 Changsha, China; 6 Translational & Clinical Research Institute, Faculty of Medical Sciences, Newcastle University, NE2 4HH Newcastle upon Tyne, UK

**Keywords:** Gestational diabetes mellitus, MiR-326, miR-532-3p, RNA-sequencing, Biomarker, Real-time quantitative PCR, Signaling pathways

## Abstract

**Purpose:**

Gestational diabetes mellitus (GDM) significantly threatens maternal and fetal health, necessitating early and accurate diagnostic tools. This study aimed to identify and validate circulating microRNAs (miRNAs) as novel biomarkers for GDM.

**Methods:**

A two-phase cross-sectional study enrolled 55 GDM patients and 55 matched healthy pregnant controls. In the discovery phase, RNA sequencing (RNA-seq) of peripheral blood RNA from a randomly selected subset (5 GDM patients, 5 controls) identified differentially expressed miRNAs. The validation phase employed reverse transcription and quantitative PCR (qPCR) in the entire cohort to confirm identified miR-326 and miR-532-3p expression. Bioinformatics analyses (Gene Ontology [GO] and Kyoto Encyclopedia of Genes and Genomes [KEGG]) investigated their functional roles using a consensus-based approach across multiple databases.

**Results:**

RNA-seq revealed significant upregulation of miR-326 (mean normalized counts = 14.59, *P* < 0.05; log_2_[Fold Change] = 0.93, *P* < 0.01) and miR-532-3p (mean normalized counts = 46.45, *P* < 0.05; log_2_[Fold Change] = 0.66, *P* < 0.01) in GDM patients, a finding corroborated by cross-referencing with the Gene Expression Omnibus (GEO) database. qPCR validation confirmed significantly higher expression for both miRNAs in GDM (*P* < 0.001), with strong negative correlations to fasting, 1-hour and 2-hour postprandial glucose levels. Receiver operating characteristic (ROC) curve analysis revealed excellent diagnostic performance (area under the curve [AUC]: 0.95 [95% CI: 0.91–0.99] for miR-326 and 0.96 [95% CI: 0.93–0.99] for miR-532-3p), robustly confirmed by bootstrap resampling. Functional analyses linked these miRNAs to phosphoinositide 3-kinase (PI3K)/Akt and Rap1 signaling pathways.

**Conclusion:**

This study provides compelling evidence for circulating miR-326 and miR-532-3p as a synergistic pair of biomarkers for GDM. Their high diagnostic accuracy and mechanistic insights into metabolic dysregulation position them as a promising complementary tool for early GDM risk assessment and for understanding GDM pathophysiology.

**Supplementary Information:**

The online version contains supplementary material available at 10.1007/s40618-026-02829-z.

## Introduction

Gestational diabetes mellitus (GDM), characterized by impaired glucose tolerance first recognized during pregnancy, is one of the most prevalent metabolic disorders affecting pregnant women worldwide [1]. The International Diabetes Federation (IDF) recently estimates that approximately 14% of pregnant women worldwide are affected by GDM [2]. In China, the prevalence is notably higher, ranging from 17.5% to 18.9% [3]. GDM poses significant risks not only to maternal health but also to fetal development and long-term health outcomes for both mother and offspring. The pathophysiology of GDM is complex, involving insulin resistance and impaired pancreatic β-cell function [1]. These metabolic disturbances can trigger a cascade of adverse effects, including increased risks of gestational hypertension, preeclampsia, and miscarriage for mothers [4], as well as macrosomia, hypoglycemia, abnormal fetal growth, and even mortality for neonates [5, 6]. Recent multi-omics investigations have begun to unravel this complexity; for instance, a systematic review of proteomics has highlighted widespread protein dysregulation in GDM [7], and specific placental proteome abnormalities have been linked to adverse fetal growth outcomes [8]. Furthermore, GDM elevates the long-term risk of metabolic syndrome, diabetes, and cardiovascular diseases for both mothers and their offspring [9]. Given the high prevalence and potential dangers of GDM, there is an urgent need for effective early predictive and diagnostic markers.

The current gold standard for GDM diagnosis is the 75 g oral glucose tolerance test (OGTT) conducted between 24 and 28 weeks of gestation [10]. However, this approach has several limitations. Firstly, it often suffers poor patient compliance due to the requirement of fasting and consuming a large amount of glucose solution. Secondly, the timing of this test may be suboptimal for early intervention, as metabolic derangements of GDM may have already impacted fetal development by this stage [10]. Evidence indicates that early screening and intervention (e.g., at 16–18 weeks gestation) can significantly reduce the risk of neonatal complications such as macrosomia and cardiomyocyte hypertrophy relative to conventional late-stage diagnosis [11]. Furthermore, studies demonstrate that diagnosing and treating GDM between 24 and 28 weeks of pregnancy can effectively control short-term maternal complications such as macrosomia, shoulder dystocia, preeclampsia, and gestational hypertension [12, 13]. This highlights the critical importance of developing complementary diagnostic biomarkers to enhance the OGTT efficacy. An optimal diagnostic biomarker for GDM should be readily obtainable, highly specific, and sensitive, facilitating early diagnosis. Previous studies have explored various biomarkers in GDM pregnancies, including Sex Hormone-Binding Globulin (SHGB), Fibroblast Growth Factor 21 (FGF-21), and Adiponectin. However, controversies regarding methods, diagnostic criteria, and treatment targets persist, and the specificity and sensitivity of these biomarkers need improvement [14–16].

In recent years, microRNAs (miRNAs) have emerged as promising candidates for early and accurate diagnosis of various metabolic disorders, including GDM. miRNAs are a class of evolutionarily conserved endogenous non-coding RNAs that regulate gene expression at the post-transcriptional level by binding to target mRNAs, participating in numerous biological processes including glucose metabolism and insulin signaling [17]. Recent literatures have demonstrated that miRNAs are dysregulated in metabolic diseases such as type 2 diabetes and obesity, contributing to pathological processes including insulin resistance and pancreatic β-cell dysfunction [18]. Several studies have reported altered miRNA expression profiles in the peripheral blood of GDM pregnant women. For example, the serum levels of miR-132-3p and miR-122-5p are significantly higher in GDM patients compared to controls, and these miRNAs are involved in the regulation of trophoblast cell proliferation, differentiation, insulin secretion, and glucose transport [19]. Additionally, the combined diagnostic performance of miR-423-5p, miR-122-5p, miR-148a-3p, miR-192-5p, and miR-99a-5p has shown superior sensitivity and specificity compared to traditional models based on fasting glucose, Body Mass Index (BMI), and age [20]. However, research on GDM-related miRNAs remains insufficient, with many differentially expressed miRNAs yet to be fully analyzed and validated for their diagnostic value. Moreover, current studies on miRNAs in GDM mostly employ targeted approaches, i.e., testing selected miRNAs, which limits the possibility of discovering new miRNAs. Non-targeted methods such as RNA-seq can generate millions of small RNA sequences from a single sample, enabling both the measurement of miRNA abundance and the discovery of novel miRNAs [21], thus providing more comprehensive molecular information for the early prediction and diagnosis of GDM.

This study employed an innovative two-phase cross-sectional design aiming to investigate the diagnostic potential of key circulating miRNAs as risk biomarkers for GDM while unraveling their mechanistic roles in disease pathogenesis. We recruited 55 GDM patients and 55 gestational age- and age-matched healthy pregnant controls, employing a cost-efficient framework that optimizes discovery and validation. In the discovery phase, RNA-seq of peripheral blood RNA from a randomly selected subset of 10 participants (5 GDM patients and 5 controls) pinpointed differentially expressed miRNAs, including miR-326 and miR-532-3p, cross-referenced with GDM-related datasets (GSE218696 and GSE192813) from the Gene Expression Omnibus (GEO) database. The validation phase assessed these miRNAs’ expression profiles and diagnostic utility across the entire cohort of 110 subjects using our in-house developed quantitative real-time PCR (qPCR) assay. This streamlined approach ensures high-value biomarker discovery with statistical rigor, offering a replicable model for resource-constrained settings.

Drawing on emerging evidence of miRNAs’ regulatory roles in metabolic disorders, we propose two scientific hypotheses: (1) specific circulating miRNAs, such as miR-326 and miR-532-3p, identified via RNA-seq and GEO dataset cross-reference and quantified through qPCR, can accurately differentiate GDM patients from healthy controls, as validated by receiver operating characteristic (ROC) curve analysis; and (2) these miRNAs modulate the pathogenesis of GDM by regulating essential pathways involved in glucose and lipid metabolism and insulin signaling, as explored through Kyoto Encyclopedia of Genes and Genomes (KEGG) and Gene Ontology (GO) analyses. The novelty of this study lies in several aspects. Firstly, to our knowledge, this is the first study to investigate the expression profiles of miR-326 and miR-532-3p in a GDM patient cohort, addressing a key knowledge gap in this field. Secondly, it utilized advanced sequencing and bioinformatics to identify GDM-related differentially expressed miRNAs, aiming to discover novel pathological markers and therapeutic targets. Thirdly, we developed a real-time fluorescent qPCR assay that integrates accessibility, cost-effectiveness, and good patient compliance, positioning this molecular diagnostic tool as a promising complement to current GDM screening approaches.

## Methods

### Study design

**Fig. 1:**
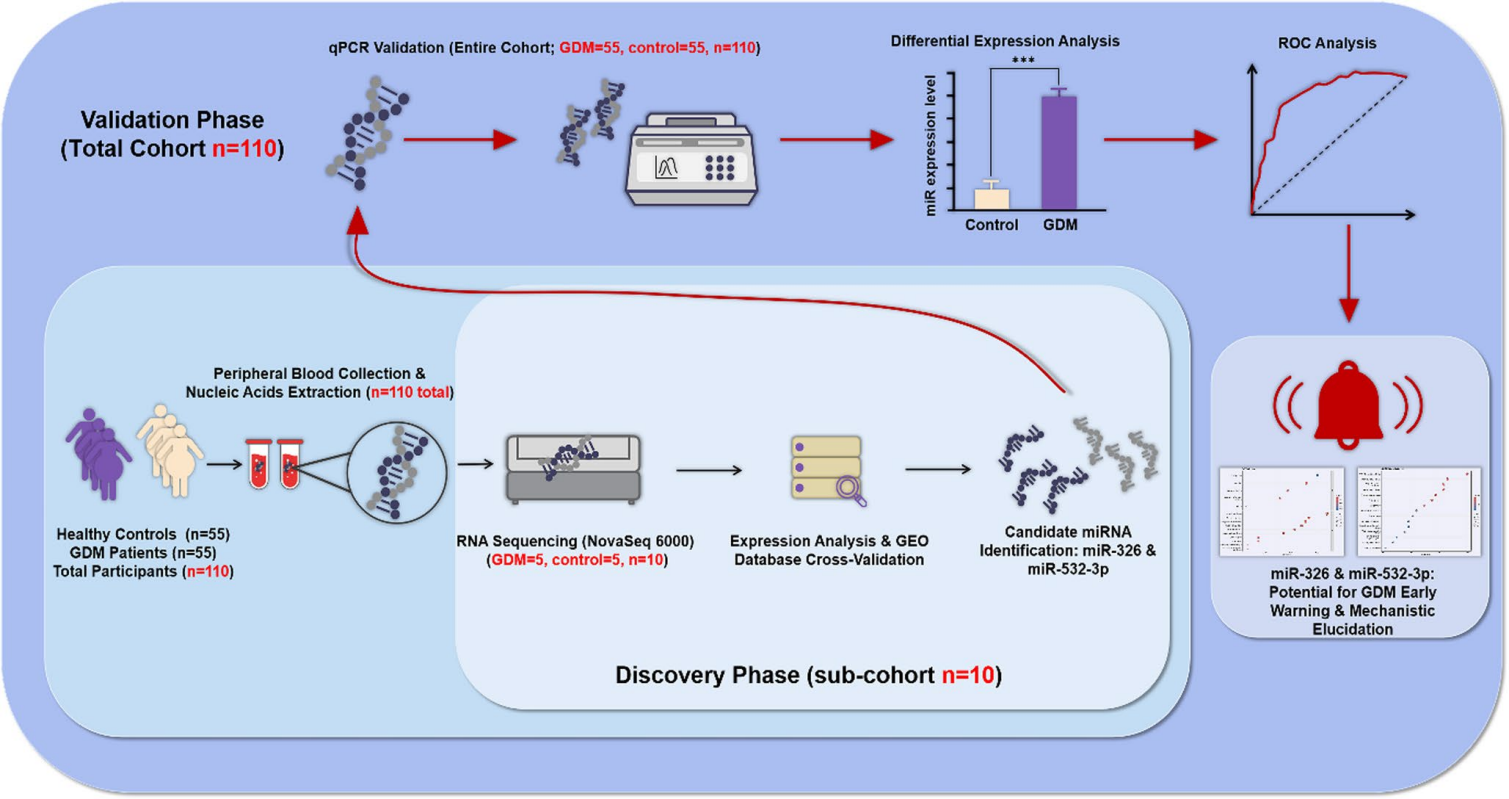
Study workflow for identifying and validating circulating miRNA biomarkers in GDM

This study was conducted using a two-phase, cross-sectional design to identify and validate circulating miR-326 and miR-532-3p as early diagnostic biomarkers for GDM. The discovery phase aimed to identify differentially expressed miRNAs using RNA-seq in a small, randomly selected cohort, The subsequent validation phase aimed to confirm the diagnostic performance of the identified miRNAs using a laboratory developed qPCR assay in the full study cohort. This study was a collaborative effort between the First Hospital of Putian and the Medicine and Biological Engineering Technology Research Centre of the Ministry of Health (MBETRC). The study design incorporated a clear division of responsibilities: the First Hospital of Putian managed patient recruitment (including both GDM and healthy control groups), clinical data collection, and peripheral blood sampling. The MBETRC was responsible for the subsequent molecular and bioinformatic analyses, including RNA extraction, library construction, RNA-seq, and comprehensive bioinformatic analyses. Additionally, the MBETRC designed reverse transcription and qPCR assays and performed these experiments to validate the RNA-seq results. The study workflow (Fig. 1) integrated RNA-seq for exploratory screening, qPCR for confirmatory detection, and bioinformatics for mechanistic insights, forming a rigorous and comprehensive experimental framework.

### Study subjects

Between June 30, 2022, and December 30, 2023, we recruited a total of 110 pregnant women, including 55 GDM patients and 55 healthy pregnant controls matched for age, gestational age, and pre-pregnancy BMI, through random sampling from the obstetrics clinic of the First Hospital of Putian. Inclusion criteria were: (1) singleton pregnancy; (2) gestational age of 24–28 weeks; (3) age between 24 and 36 years. Exclusion criteria included: (1) history or family history of type 1 or type 2 diabetes, history or family history of hypertension, cardiovascular disease, or other endocrine and metabolic diseases (including thyroid disease); (2) presence of other pregnancy complications such as preeclampsia or placental abruption; (3) multiple pregnancies or severe fetal malformations; (4) current smoking; (5) use of medications affecting insulin sensitivity or glucose metabolism (e.g., metformin, steroids) within the past three months. GDM was diagnosed according to the 2021 American Diabetes Association (ADA) criteria [22]: a 75 g OGTT with venous blood glucose levels measured at fasting and at 1 and 2 h post-ingestion. GDM was diagnosed if any of the following thresholds were met: fasting ≥ 5.1 mmol/l, 1 h ≥ 10.0 mmol/l, 2 h ≥ 8.5 mmol/l. Eligible GDM patients meeting the inclusion and exclusion criteria and control pregnant women with normal OGTT results were identified during consultation and recruited upon expressed willingness to participate. The discovery cohort was composed of 10 participants (5 GDM and 5 controls) randomly selected from the total cohort using a random number generator to ensure unbiased representation. The remaining participants, along with the discovery subset, formed the validation cohort. All participants provided written informed consent, and the study was approved by the Research Ethics Committees of the First Hospital of Putian and MBETRC (approval number 2022027; for detail, please refer to the Biosafety and Ethics Statement in the Declarations section).

### Sample collection, processing and RNA extraction

Fasting peripheral venous blood (5 ml) was collected from each of the 110 participants in the morning and placed in EDTA anticoagulant tubes. Samples were transported to the MBETRC within 48 h of collection, following strict national biological material transport regulations. Samples were packaged appropriately and kept at 4 °C during transportation. Within 4 h of arrival, samples were processed by centrifugation at 1500 ×g for 15 min at 4 °C to separate the plasma. Plasma RNA was extracted using the Trizol method, following protocols described in a prior publication [23]. RNA concentration and purity were assessed using the Nanodrop 2000 spectrophotometer (Thermo-Fisher Scientific, Waltham, MA). Acceptable RNA purity was defined by an optical density (OD)260/280 ratio of 1.7–2.5 and an OD260/230 ratio of 0.5–2.5. RNA integrity was evaluated using the Agilent 2100 Bioanalyzer (Agilent Technologies, Santa Clara, CA), with acceptable RNA integrity defined by an RNA Integrity Number (RIN) ≥ 8 and a 28 S/18S ratio ≥ 1.5.

### Discovery phase: RNA library Preparation and sequencing

In the discovery phase, 1 µg of freshly extracted total RNA from each of the 10 randomly selected GDM and control participants was used for library construction using the VAHTS Small RNA Library Prep Kit for Illumina (Vazyme Biotech Co., Ltd, Nanjing, China), strictly following the manufacturer’s instructions.

Upon completion of library construction, preliminary quantification was performed using the Qubit 3.0 fluorometer (Thermo-Fisher Scientific, Waltham, MA). The libraries were diluted to a final concentration of 1.5 ng/µl, and 1 µl of each library was analyzed using the Agilent 2100 Bioanalyzer. An acceptable miRNA library ranged between 143 bp (bp) and 147 bp.

Sequencing was conducted on the Illumina NovaSeq 6000 platform (Illumina, San Diego, CA) using the NovaSeq 6000 Reagent Kits (Illumina, San Diego, CA), employing the paired-end 150 sequencing strategy. The sequencing principle involves synthesis-by-sequencing, as detailed elsewhere [24].

### Sequencing data processing

Internal Perl scripts were used to process the raw reads in FASTQ format, removed reads containing adapters, reads with N (indicating undetermined bases), and low-quality reads. Sequences shorter than 18 nt or longer than 30 nt were also filtered out, ultimately obtaining clean reads. All downstream analyses were based on these high-quality clean reads.

The clean reads were aligned against the Silva, GtRNAdb, Rfam, and Repbase databases using the Bowtie Tools software, to filter out ribosomal RNA, transfer RNA, small nuclear RNA, and small nucleolar RNA, as well as other repetitive sequences. Subsequently, the remaining reads were aligned to the reference genome Homo_sapiens.GRCh38_release95 for sequence alignment and analysis. Known miRNAs were identified by aligning the mapped reads to the mature miRNA sequences in the miRBase database, allowing for a maximum of one mismatch, and considering a range of 2 nt upstream and 5 nt downstream of each mature miRNA sequences. Reads identified in this manner were considered as known miRNAs.

Normalization was conducted using the VOOM function in the limma package of R software. Differentially expressed miRNAs were also identified using the limma package in R software as described previously [25]. The selection criteria were set at |log_2_(Fold Change [FC])| ≥ 0.58 and P value ≤ 0.05, yielding 229 differentially expressed miRNAs. To select candidates for validation, a data-driven multi-criteria approach was applied: (1) initial prioritization of top-10 by fold-change, refined by literature review for metabolic relevance; (2) target prediction for all 229 using TargetScan, miRDB, and DIANA-microT 2023, with consensus intersections; (3) GO/KEGG enrichment on targets, favoring GDM-related pathways (e.g., PI3K/Akt, insulin signaling); (4) cross-validation in independent GEO datasets (GSE218696, GSE192813); and (5) preliminary ROC analysis (the area under the curve [AUC] > 0.85 in discovery subset). This framework ensured selection of miR-326 and miR-532-3p based on integrated biological and diagnostic potential. P values from the initial hypothesis tests were adjusted using the Benjamini-Hochberg correction method, with the false discovery rate as the key indicator for selecting differentially expressed miRNAs. Additionally, datasets GSE218696 and GSE192813 were downloaded from the GEO database, merged, and analyzed using the same criteria to cross-validate candidate miRNAs.

### GO and KEGG analyses

Target genes of miR-326 and miR-532-3p were predicted using TargetScan Release 8.0 (https://www.targetscan.org/vert_80/) [26], miRDB (https://mirdb.org/), and DIANA-microT 2023 (https://dianalab.e-ce.uth.gr/microt_webserver/#/). TargetScan predicts miRNA biological targets by searching for conserved 8-mer, 7-mer, and 6-mer sites that match the seed region of each miRNA [26]. miRDB provides target predictions and functional annotations using MirTarget, an algorithm developed from analyzing thousands of miRNA-target interactions in high-throughput sequencing experiments [27]. DIANA-microT 2023 provides computationally predicted miRNA-gene interaction data [28]. A high-confidence consensus list of target genes was generated by taking the intersection of the prediction lists from all three databases. This consensus list was visualized using a Venn diagram (created with the ggVennDiagram R package, version 1.12) (Supplementary Figure S2) and used for all subsequent GO and KEGG analyses.

Subsequently, GO and KEGG enrichment analyses were performed on the target genes using the ClusterProfiler package (version 4.12.0) in R. GO enrichment analysis identified functional categories within biological processes, cellular components, and molecular functions, with an adjusted P value ≤ 0.05 and q value ≤ 0.2 considered significant, and results were visualized. KEGG enrichment analysis focused on metabolic and signaling pathways, with significant thresholds set at *P* ≤ 0.05 and q value ≤ 0.2, and enrichment pathway diagrams were plotted.

### cDNA synthesis

For cDNA synthesis, freshly extracted RNA from all 110 participants was reverse-transcribed. The reverse transcription mixture consisted of the following components: 500 nM dNTPs, 2 µl of 10X RT buffer, 300 nM specific primer, 200 ng of total RNA, 40 U MMLV reverse transcriptase, 12 U RNase inhibitor (all purchased from Thermo-Fisher Scientific, Waltham, MA), and RNase-free water to a final volume of 20 µl. The mixture was combined with RNA and subjected to reverse transcription using the Gene Amp PCR system 9700 (Thermo-Fisher Scientific, Waltham, MA) under the following conditions: 16°C for 30 min; 42°C for 40 min; 85°C for 5 min. The specific primer sequences were as follows: U6 primer sequence 5’-CGCTTCACGAATTTGCGTGTCAT-3’, miR-326 primer sequence 5’- GTCGTATCCAGTGCGTGTCGTGGAGTCGGCAATTGCACTGGATACGACCTGGAG-3’, miR-532-3p primer sequence 5’-GTCGTATCCAGTGCGTGTCGTGGAGTCGGCAATTGCACTGGATACGACTGCAAGC-3’.

### Validation phase: qPCR detection

The expression levels of miR-326, miR-532-3p, and the internal reference gene U6 in various samples were quantified using qPCR. The sequences of the respective primers used are listed in Table 1. The qPCR reactions were carried out using the Arraystar SYBR^®^ Green real-time PCR Master Mix (Arraystar Inc., Rockville, MD). The reaction mixture included the following components: 5 µl 2 × Master Mix, 500 nM forward primer, 500 nM reverse primer, 2 µl of the template cDNA and diethylpyrocarbonate-treated water for a total reaction volume of 10 µl. The qPCR was performed using the QuantStudio 5 Real-time PCR System (Thermo-Fisher Scientific, Waltham, MA) with the following thermal cycling conditions: 95 °C for 10 min; followed by 40 cycles of 95 °C for 10 s and 60 °C for 60 s. A Cycle threshold (Ct) value below 38 is considered the threshold for positivity. The relative expression levels of miRNAs were quantified using the 2^∆∆Ct^ method as detailed in a prior publication [29]. Data accuracy was ensured by running triplicates for each sample. Each qPCR run included a positive control, a template-free negative control as well as a reverse-transcribed free negative control. Results were analyzed and visualized using GraphPad PRISM 8 software.

**Table 1:** Primers sequences used in quantitative PCR assay

Gene	Primer name	Function	Sequence (5’-3’)
U6	U6-F	Internal Reference	5’- GCTTCGGCAGCACATATACTAAAAT − 3’
	U6-R		5’- CGCTTCACGAATTTGCGTGTCAT − 3’
miR-326	miR-326-GSPF^*^	Target miRNA	5’- GTAAAACCTCTGGGCCCTTC − 3’
	miR-326-GSPR^*^		5’- GTGCGTGTCGTGGAGTCG − 3’
miR-532-3p	miR-532-3p-GSPF^*^	Target miRNA	5’- GGAACCTCCCACACCCAAG − 3’
	miR-532-3p-GSPR^*^		5’- GTGCGTGTCGTGGAGTCG − 3’

^*^GSP, Gene-specific primer. GSPs were employed for the precise amplification of cDNA derived from miRNAs of interest. In this study, these GSPs were designed to specifically target the miRNA sequences following reverse transcription. The reverse primers of the GSPs were particularly designed to match the downstream region of the reverse transcription primers, ensuring specific and efficient amplification of the target miRNAs

### Biochemical analysis

Clinical biochemical indicators, including triglycerides (TG), cholesterol (CHO), and high-density lipoprotein (HDL), were analyzed in the clinical laboratory of the First Hospital of Putian with an additional blood sample using the Toshiba TBA-2000FR biochemical analyzer (Toshiba Medical Systems Corporation, Ōtawara, Japan). The assays were performed with reagent kits provided by Beijing Leadman Biochemistry Co., Ltd. (Beijing, China) according to the manufacturer’s instructions. Specifically, the glycerol-3-phosphate oxidase-para-aminophenazone (GPO-PAP) method for TG, the cholesterol oxidase-peroxidase (CHOD-POD) method for CHO, and the high-density lipoprotein cholesterol (HDL-C) direct assay for HDL were employed. All measurements were performed with proper internal quality control samples added in the same run in strict accordance with standardized protocols to ensure the accuracy and reliability of the results.

### Statistical analysis

Data analysis was performed using R software version 4.3. Continuous variables following a normal distribution were presented as mean ± standard deviation (x ± s) and compared between groups using the t-test. Non-normally distributed data were presented as median (interquartile range) and analyzed using the Wilcoxon rank-sum test. Spearman’s correlation analysis was conducted to assess the correlation between miRNA expression levels and baseline data, with the results visualized in a correlation coefficient matrix annotated with statistical significance. ROC curve analysis was employed to evaluate the diagnostic efficacy of miR-326 and miR-532-3p for GDM risk in the 110-participant validation cohort, with the AUC approaching 1.0 indicating high diagnostic performance and values at or below 0.5 indicating low utility. Diagnostic performance metrics were calculated as follows: sensitivity = true positives / (true positives + false negatives); specificity = true negatives / (true negatives + false positives); accuracy = (true positives + true negatives) / total sample size; positive predictive value = true positives / (true positives + false positives); negative predictive value = true negatives / (true negatives + false negatives). In the ROC curve analysis, positive threshold values for miR-326 and miR-532-3p were determined using Youden’s Index, calculated as: Youden’s Index = Sensitivity + Specificity − 1. The threshold corresponding to the maximum Youden’s Index was selected as the optimal positive cutoff point, which maximizes the balance between sensitivity and specificity for diagnostic purposes. To evaluate the robustness of the diagnostic performance of miR-326 and miR-532-3p, we conducted a sensitivity analysis using 1000 bootstrap resampling iterations. In this process, data were randomly sampled with replacement from the original dataset to generate new datasets, and ROC curves were constructed for each resampled dataset. The AUC values from these 1000 iterations were analyzed to assess the stability and reliability of the diagnostic performance metrics. A P value < 0.05 was considered statistically significant.

## Results

### Baseline characteristics of study participants

Baseline demographic data and clinical biochemical data were collected and analyzed for the 110 participants (55 GDM patients and 55 matched healthy controls), as summarized in Table 2. The groups were successfully matched for maternal age, gestational age, pre-pregnancy weight, pre-pregnancy BMI, height, and cholesterol. There were also no significant differences in post-pregnancy weight or post-pregnancy BMI between the groups (*P* > 0.05). Notably, an intra-group analysis revealed that both the GDM and control groups experienced a statistically significant increase in weight and BMI during pregnancy (*P* < 0.05 for all), as detailed in Supplementary Table 1. As expected, significant metabolic differences characteristic of GDM were observed. Specifically, the GDM cohort exhibited significantly higher levels of fasting glucose, 1-hour and 2-hour postprandial glucose, and TG, along with significantly lower levels of HDL, compared to the control group (all *P* < 0.05). These data confirm the distinct metabolic dysregulation present in the GDM participants and establish a well-characterized cohort for biomarker analysis.

**Table 2:** Baseline characteristics of the study participants

Variable	GDM Group (*N* = 55)	Control Group (*N* = 55)	*P* Value
Age (years)	29.71 ± 3.48	28.69 ± 4.16	0.17
Fasting glucose (mmol/l)	5.35 (0.99)	4.38 (0.29)	< 0.01
1-hour postprandial glucose (mmol/l)	9.88 ± 2.12	7.26 ± 1.62	< 0.01
2-hour postprandial glucose (mmol/l)	8.30 ± 1.69	6.38 ± 0.89	< 0.01
Height (cm)	161.13 ± 4.39	160.69 ± 5.12	0.63
Pre-pregnancy weight (kg)	57.00 (13.25)	55.00 (9.50)	0.28
Pre-pregnancy BMI (kg/m^2^)	21.64 (4.01)	21.23 (3.96)	0.24
Post-pregnancy weight (kg)	57.76 (9.04)	56.16 (9.05)	0.36
Post-pregnancy BMI (kg/m^2^)	22.23 (3.28)	21.73 (3.26)	0.43
Triglycerides (mmol/)	1.38 (0.78)	0.95 (0.59)	0.01
High-density lipoprotein (mmol/L)	1.76 ± 0.29	1.92 ± 0.36	0.01
Cholesterol (mmol/l)	4.56 (0.65)	4.33 (0.85)	0.46

### Screening and identification of differentially expressed miRNAs

**Fig. 2:**
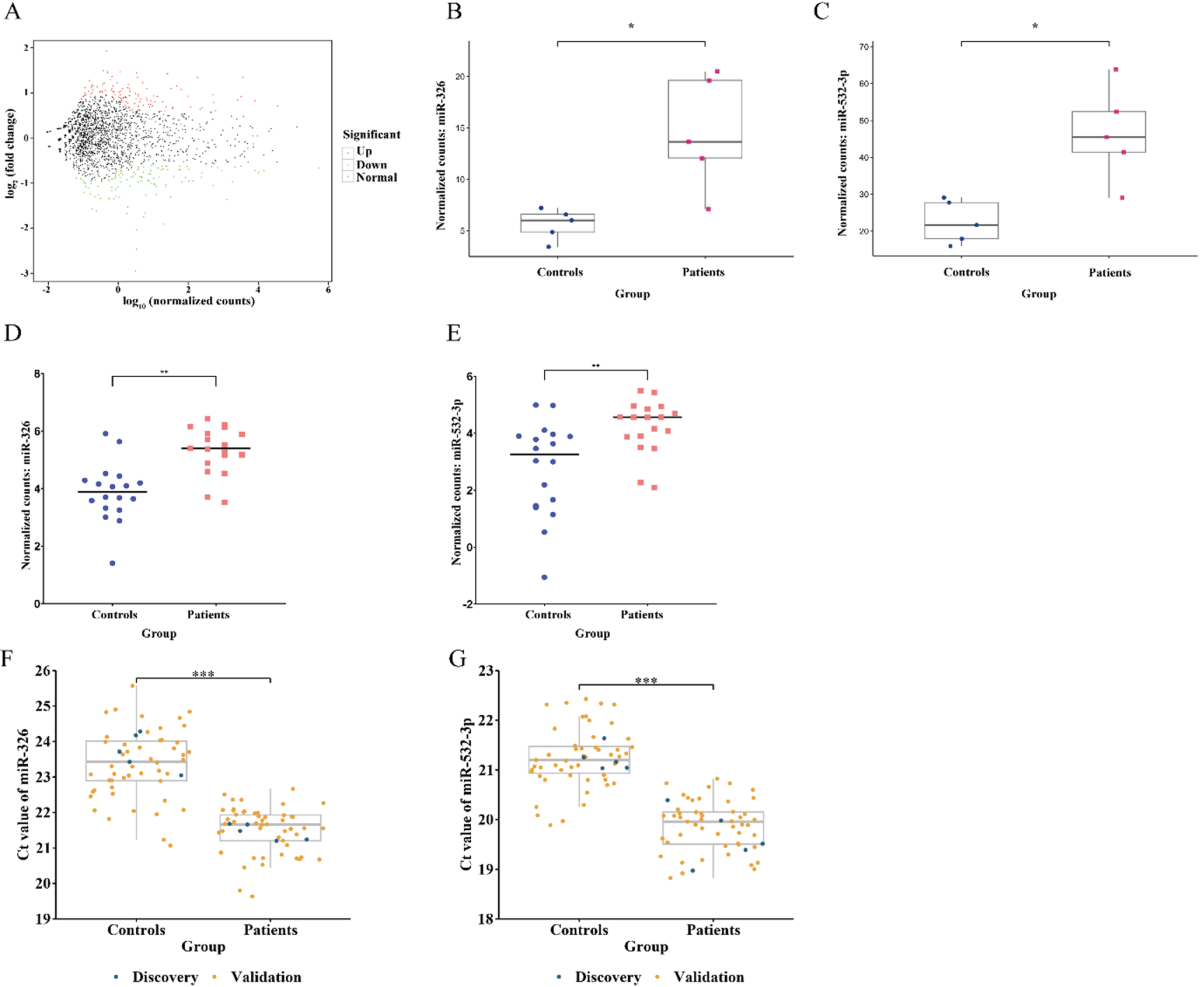
Differential expression and validation of miRNAs in GDM patients compared to controls.(**A**) M-versus-A (MA) plot illustrating the differential expression of miRNAs between GDM patients and control subjects. Significantly upregulated miRNAs are highlighted in red, while downregulated miRNAs are shown in green. (**B**) and (**C**) depict the comparative expression levels of miR-326 and miR-532-3p between GDM patients and controls, respectively, demonstrating significant upregulation in the GDM group (**: *P* < 0.01). (**D**) and (**E**) validate the expression patterns of miR-326 and miR-532-3p using publicly available datasets GSE218696 and GSE192813, confirming consistent upregulation in GDM patients (**: *P* < 0.01). (**F**) and (**G**) further corroborate these findings, demonstrating significantly lower Ct values for miR-326 and miR-532-3p in the GDM group via quantitative PCR analysis (***: *P* < 0.001)

Differential expression analysis of high-quality transcriptome sequencing data from peripheral blood samples identified miRNAs with significantly different expression levels between the GDM and control groups. Using stringent criteria (|log_2_(FC)| ≥ 0.58 and P value ≤ 0.05), we identified that a total of 229 miRNAs showed significant differential expression, with 114 upregulated and 115 downregulated in the GDM group compared to controls (Fig. 2A, Supplementary Table 2). To identify the most clinically relevant candidates from this list, we applied a rigorous, data-driven process: initial fold-change ranking was supplemented by comprehensive target prediction and GO/KEGG analyses across all candidates, prioritizing enrichment in metabolic pathways (e.g., PI3K/Akt for glucose regulation). Cross-validation in the merged GEO dataset confirmed consistent upregulation (miR-326 log_2_(FC) = 1.475, *P* < 0.001; miR-532-3p log_2_(FC) = 1.482, *P* = 0.004), and preliminary ROCs showed strong discriminatory power (AUC = 0.95–0.96). These miRNAs also exhibited expression synergy (Ct correlation *r* = 0.63, *P* < 0.001 in validation), underscoring their dual-biomarker potential.

Among these differentially expressed miRNAs, miR-326 and miR-532-3p emerged as outstanding candidates that satisfied both criteria. Through analysis of RNA-seq data, we found that the expression levels of miR-326 (mean normalized counts = 14.59, *P* < 0.05; log_2_[FC] = 0.93, *P* < 0.01) and miR-532-3p (mean normalized counts = 46.45, *P* < 0.05; log_2_[FC] = 0.66, *P* < 0.01) were significantly higher in the GDM group compared to the control group (Fig. 2B and C). To validate this finding, we analyzed the expression levels of miR-326 and miR-532-3p in the publicly available datasets GSE218696 and GSE192813. The results showed that these two miRNAs also exhibited significant expression differences between the GDM and control groups (*P* < 0.01), consistent with the trend observed in our study cohort (Fig. 2D and E).

In addition to the validation using public datasets, we further performed qPCR validation in the full 110-participant study cohort. qPCR analysis of peripheral blood RNA samples from the GDM and control groups revealed that the expression levels of miR-326 and miR-532-3p were significantly higher in the GDM group compared to the control group (*P* < 0.001, Fig. 2F and G). This further confirmed the expression difference trends observed in our miRNA sequencing and public datasets.

### Correlation analysis of miR-326 and miR-532-3p with clinical biochemical indicators

**Fig. 3:**
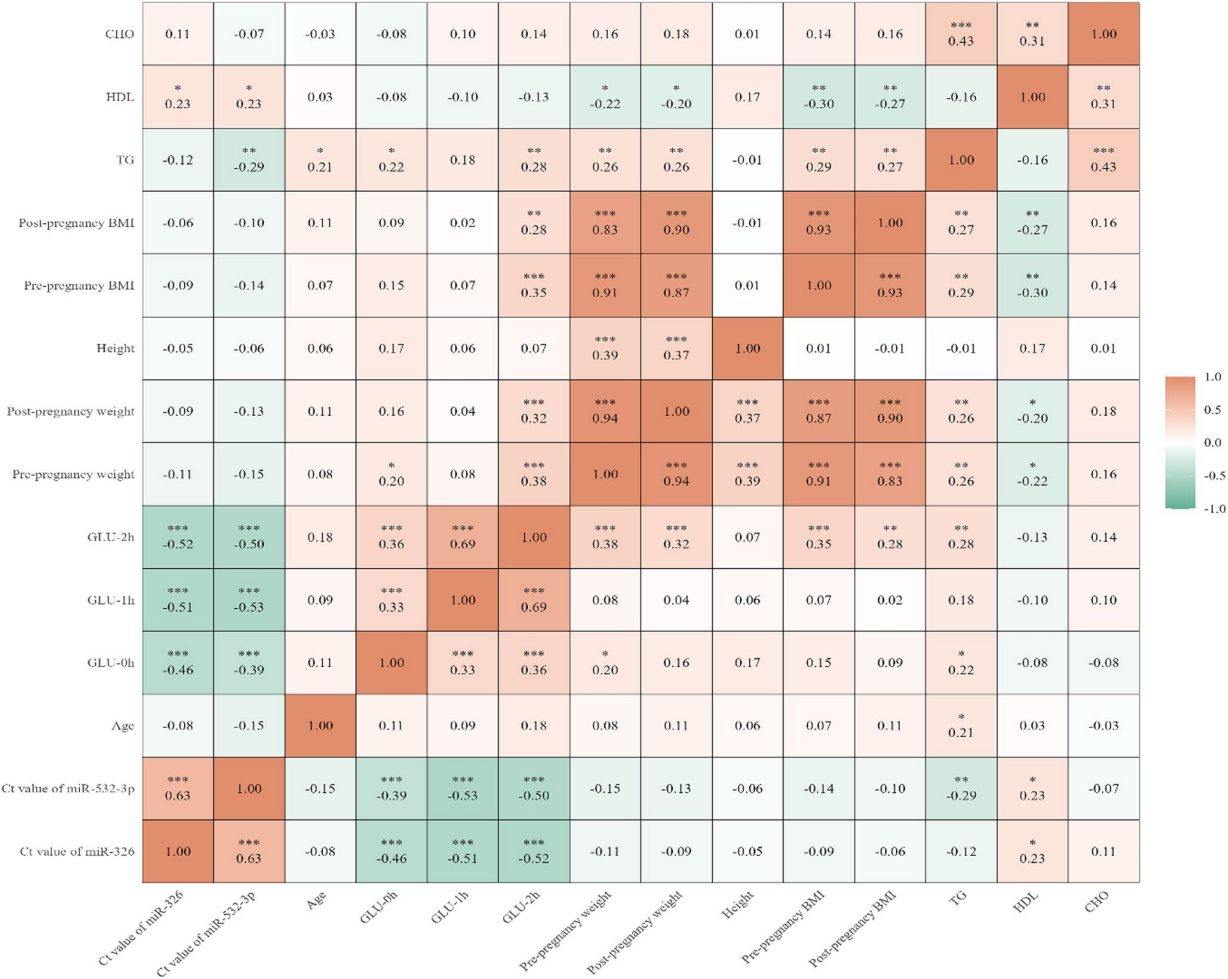
Spearman correlation matrix. This heatmap illustrates the Speazrman correlation coefficients between various clinical and biochemical parameters in patients with GDM and control subjects. Variables include fasting glucose (GLU-0 h), 1-hour postprandial glucose (GLU-1 h), 2-hour postprandial glucose (GLU-2 h), triglycerides (TG), high-density lipoprotein (HDL), cholesterol (CHO), age, pre-pregnancy BMI, height, and pre-pregnancy weight, as well as Ct values of miR-326 and miR-532-3p. Positive correlations are indicated by orange shading, while negative correlations are indicated by green shading. The intensity of the color represents the strength of the correlation, with deeper colors indicating stronger correlations. Statistical significance is denoted by * *P* < 0.05, **P < 0.01 and ***P < 0.001

Following their identification as differentially expressed miRNAs in the discovery phase (*n* = 10), we evaluated the associations of miR-326 and miR-532-3p with clinical biochemical indicators in the entire cohort of 110 participants using Spearman’s correlation analysis. The qPCR Ct values of miR-326 and miR-532-3p exhibited a significant positive correlation (*r* = 0.63, *P* < 0.001). Both miRNAs showed strong negative correlations with fasting blood glucose (miR-326: *r* = -0.46, *P* < 0.001; miR-532-3p: *r* = -0.39, *P* < 0.001), 1-hour postprandial glucose (miR-326: *r* = -0.51, *P* < 0.001; miR-532-3p: *r* = -0.53, *P* < 0.001), and 2-hour postprandial glucose (miR-326: *r* = -0.52, *P* < 0.001; miR-532-3p: *r* = -0.50, *P* < 0.01). Furthermore, the analysis revealed additional associations with metabolic health indicators. Both miRNAs’ Ct values were positively correlated with HDL (*r* = 0.23, *P* < 0.05), whereas only miR-532-3p’s Ct value showed a negative correlation with TG (*r* = -0.29, *P* < 0.01). Additionally, 2-hour postprandial glucose levels were positively correlated with pre-pregnancy weight (*r* = 0.348, *P* < 0.001), post-pregnancy weight (*r* = 0.32, *P* < 0.001), pre-pregnancy BMI (*r* = 0.35, *P* < 0.001), and post-pregnancy BMI (*r* = 0.28, *P* < 0.01) (Fig. 3). These findings highlight the complex relation between circulating miRNAs and metabolic dysregulation in GDM.

### Diagnostic analysis of miR-326 and miR-532-3p for GDM risk

**Fig. 4:**
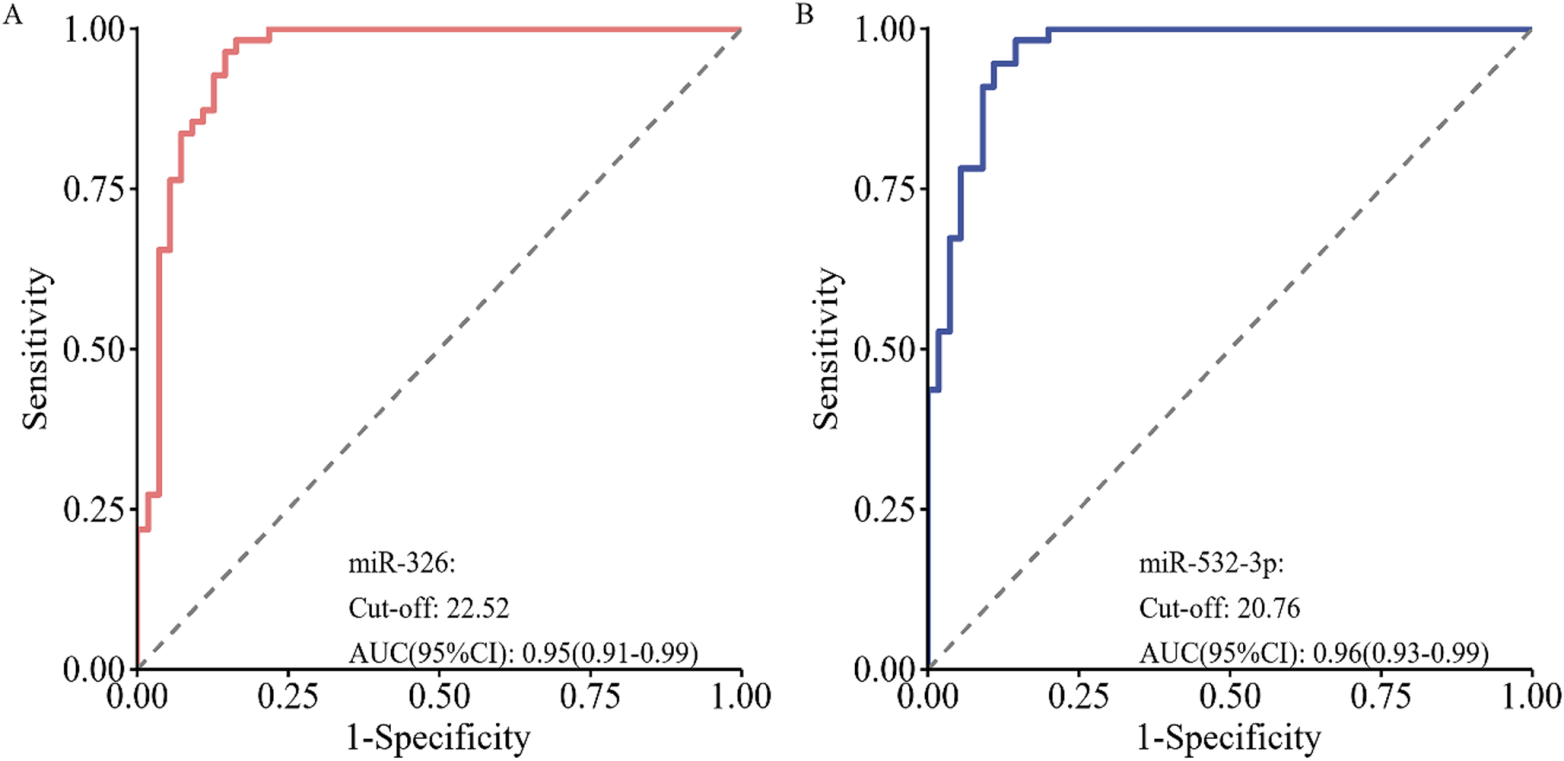
Receiver operating characteristic (ROC) curves for miRNA-based diagnosis of GDM risk.(**A**) ROC curve for miR-326 using qPCR Ct values, with a cut-off value of 22.52, illustrating its predictive performance for GDM risk. (**B**) ROC curve for miR-532-3p using qPCR Ct values, with a cut-off value of 20.76, demonstrating its predictive efficacy for GDM risk

To assess the clinical screening potential of miR-326 and miR-532-3p as diagnostic markers, we performed ROC curve analysis on their qPCR Ct values from the full 110-participant validation cohort. Applying Youden’s Index, optimal thresholds were established at 22.52 for miR-326 (sensitivity: 0.98, 95% CI: 0.95–1.00; specificity: 0.84, 95% CI: 0.74–0.93) and 20.76 for miR-532-3p (sensitivity: 0.98, 95% CI: 0.95–1.00; specificity: 0.86, 95% CI: 0.76–0.95) (Table 3). In our study population, Ct values below these thresholds were strongly associated with elevated GDM risk. The AUC for miR-326 in distinguishing GDM patients from healthy controls was 0.95 (95% CI: 0.91–0.99). For miR-532-3p, the performance was similarly excellent, with an AUC of 0.96 (95% CI: 0.93–0.99) (Fig. 4). Sensitivity analysis with 1000 bootstrap resampling iterations confirmed the consistency of these AUC values (Supplementary Figure S1), supporting the robustness of our findings. Collectively, these results provide strong, validated evidence that both miR-326 and miR-532-3p hold significant clinical potential as robust and highly accurate biomarkers for GDM.

**Table 3:** Diagnostic performance corresponding to different positive thresholds for miR-326 and miR-532-3p

miRNA	Positive thresholds	Sensitivity [% (95% CI)]	Specificity [% (95% CI)]	Positive predictive value [% (95% CI)]	Negative predictive value [% (95% CI)]
miR-326	22.52	0.98 (0.95-1.00)	0.84 (0.74–0.93)	0.86 (0.77–0.94)	0.98 (0.94-1.00)
miR-532-3p	20.76	0.98 (0.95-1.00)	0.86 (0.76–0.95)	0.87 (0.79–0.95)	0.98 (0.94-1.00)

### Target analysis of miR-326 and miR-532-3p

**Fig. 5:**
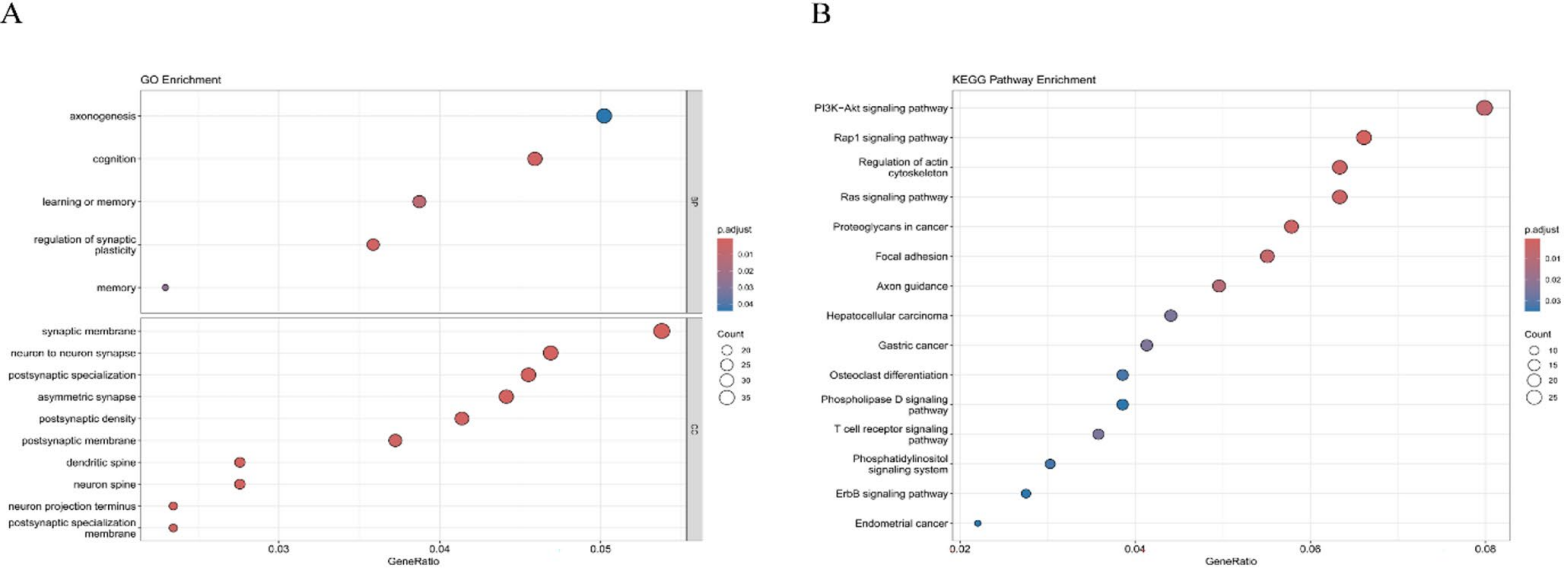
Gene Ontology (GO) and Kyoto Encyclopedia of Genes and Genomes (KEGG) pathway analysis of miR-326 and miR-532-3p target genes.(**A**) GO analysis of biological processes and cellular components targeted by the high-confidence consensus list of miR-326 and miR-532-3p target genes. The y-axis lists the enriched biological processes, and the x-axis shows the gene ratio, representing the proportion of target genes involved in each process. (**B**) KEGG pathway analysis of miR-326 and miR-532-3p target genes. The y-axis lists the enriched pathways, and the x-axis shows the gene ratio. The color gradient indicates the adjusted p-values, with red representing higher significance and blue representing lower significance. The size of the circles represents the count of genes involved in each process or pathway

After determining the diagnostic value of miR-326 and miR-532-3p, we further explored their biological functions in GDM and predicted their potential targets. Using the consensus-based approach intersecting the TargetScan, miRDB, and DIANA-microT 2023 databases, we performed target gene prediction for miR-326 and miR-532-3p and identified a high-confidence consensus list of 182 target genes (Supplementary Figure S2). Subsequently, through GO and KEGG analyses, we thoroughly investigated the primary functions of these target genes. GO analysis indicated that the high-confidence consensus target genes of miR-326 and miR-532-3p were significantly enriched in biological processes such as axonogenesis, cognition, learning and memory, and regulation of synaptic plasticity, and cellular components such as synaptic membrane, neuron to neuron synapse, post-synaptic specialization, and asymmetric synapse (Fig. 5A). KEGG pathway analysis results revealed that these target genes were predominantly involved in signaling pathways related to glucose and lipid metabolism and insulin secretion, such as the phosphoinositide 3-kinase (PI3K)/Akt signaling pathway and the Rap1 signaling pathway (Fig. 5B). These findings suggest that miR-326 and miR-532-3p play important roles in the development and progression of GDM.

## Discussion

GDM represents a significant and growing challenge to maternal and fetal health. As early diagnosis is crucial for preventing adverse neonatal outcomes [11], there is a strong demand for early and accurate diagnostic biomarkers to complement the gold-standard OGTT. The conventional OGTT is typically conducted in the later stages of pregnancy and can impose significant patient burden [2, 5, 9]. With the continuous progress in biomarker research, miRNAs have emerged as valuable research targets [18]. This case-control study employed a robust, two-phase discovery and validation approach to profile miRNAs in peripheral blood samples from pregnant women in both the GDM and control groups within a well-characterized cohort of 110 participants. The baseline metabolic aberrations in GDM (e.g., hyperglycemia, dyslipidemia) align with global cohorts [30, 31]. In the discovery phase, an in-depth RNA-seq analysis of a randomly selected subset of 10 matched GDM-control subjects, alongside simultaneous analysis of publicly available datasets, revealed two circulating miRNAs (miR-326 and miR-532-3p) to be significantly upregulated in the GDM group (*P* < 0.01). Subsequent qPCR validation across the entire cohort confirmed their overexpression (*P* < 0.001), with strong negative correlations to fasting blood glucose, and 1-hour and 2-hour postprandial glucose levels (r ranging from − 0.53 to -0.39, *P* < 0.001). These miRNAs exhibited high diagnostic accuracy (AUC: 0.95 for miR-326, 0.96 for miR-532-3p; 95% CIs: 0.91–0.99, 0.93–0.99), validated by 1000 bootstrap iterations. Insulin resistance and impaired pancreatic β-cell function are the major causes of abnormal blood glucose levels.

Research has demonstrated that multiple miRNAs are associated with insulin resistance. Studies utilizing diabetic mouse models have shown that miR-19a targets and downregulates PTEN expression, thereby influencing glycogen synthesis [32]. Similarly, overexpression of miR-29a in primary hepatocytes and mouse liver tissue reduces the protein levels of PGC-1α and G6Pase, consequently decreasing hepatic gluconeogenesis and overall glucose production [33]. Furthermore, Trajkovski et al. employed obese mouse models to identify caveolin-1 as a direct target of miR-103 and miR-107; inhibition of these microRNAs in adipocytes resulted in increased caveolin-1 expression, which enhanced and stabilized insulin receptor signaling, thereby improving insulin sensitivity [34]. Additionally, miR-96 expression is decreased in women with GDM, and functional assays confirm that miR-96 enhances β-cell function by directly targeting and downregulating PAK1 expression [35].Through RNA-seq and qPCR analyses, we assessed the expression profiles of miR-326 and miR-532-3p and observed a notable positive correlation between their Ct values. Additionally, both miRNAs exhibited a significant negative correlation with fasting blood glucose, 1-hour and 2-hour postprandial glucose levels, suggesting that their overexpression may contribute to dysregulated glycemia among GDM patients. This further indicates that these miRNAs may play a role in glucose metabolism and insulin signaling pathways, which are central to GDM pathophysiology.

Our study identifies and validates miR-326 and miR-532-3p as a synergistic pair of biomarkers exhibiting outstanding diagnostic accuracy for GDM. While recent studies have independently reported the dysregulation of these miRNAs in GDM [36, 37], our study advances the field by establishing a robust clinical validation framework that distinguishes it from prior findings. Rather than merely identifying these alterations, our study provides three distinctive contributions. First, we demonstrate superior diagnostic performance with validated AUC values of 0.95 (miR-326) and 0.96 (miR-532-3p) in a strictly matched cohort, confirmed by rigorous bootstrap analysis. Second, we uniquely characterize a synergistic co-regulation between these two miRNAs (*r* = 0.63, *P* < 0.001), suggesting they function as a coordinated regulatory module rather than isolated markers. Third, by employing a comprehensive three-layer design, spanning unbiased RNA-seq discovery, cross-validation against public GEO datasets, and large-scale qPCR verification, combined with a consensus-based bioinformatic approach, we provide the first evidence that this dual-miRNA signature mechanistically converges on the PI3K/Akt and Rap1 metabolic pathways.

The clinical utility of these miRNAs is further underscored when compared to traditional biochemical markers. For example, established markers such as leptin [38], retinol-binding protein 4 (RBP4) [39], and Fetuin-A [40] typically exhibit diagnostic specificities of 64.2%, 79.1%, and 76.2%, respectively. In contrast, miRNAs such as miR-16-5p, miR-17-5p, and miR-20a-5p have shown specificities exceeding 95% [41], demonstrating the tremendous potential of these miRNA biomarkers. Our identified miRNAs demonstrated superior specificities of 84% and 86%, alongside an exceptional sensitivity of 98%. Methodologically, our study adopted a strategic “tiered discovery-validation” framework. While RNA-seq offers powerful high-throughput screening capabilities, its high cost and complexity can hinder routine clinical application. By utilizing RNA-seq on a representative subset for unbiased discovery, followed by targeted qPCR validation across the full cohort, we balanced exploratory depth with statistical rigor. This cost-efficient design not only ensures the reliability of the identified biomarkers but also demonstrates a scalable workflow suitable for widespread clinical deployment, particularly in resource-constrained healthcare settings.

Recent literatures have elucidated that the involvement of miRNAs in modulating glucose and lipid metabolism. For example, overexpression of miR-130a, miR-130b, and miR-152 reduces the levels of glucokinase and pyruvate dehydrogenase E1 subunit-α1, thereby decreasing insulin synthesis and secretion [42]. Beyond diagnosis, our bioinformatic analyses reveal valuable insights into the potential regulatory roles of miR-326 and miR-532-3p in GDM pathogenesis. Through GO and KEGG pathway analyses, we thoroughly explored the target genes of miR-326 and miR-532-3p. The enrichment of target genes in the PI3K/Akt signaling pathway is particularly noteworthy. The PI3K/Akt pathway is recognized as a crucial pathway and plays essential roles in various physiological processes including glucose homeostasis, lipid metabolism, and protein synthesis [43–45]. Dysregulation of this pathway can lead to metabolic diseases such as obesity and type 2 diabetes [46]. While previous studies have implicated that downregulation of other miRNAs, such as miR-22 and miR-372, induces GDM through the PI3K/Akt pathway [47], our study suggests a potential link between miR-326 and miR-532-3p and this critical signaling cascade in the context of GDM. Furthermore, the Ct values of these two miRNAs in the entire 110-participant pregnant cohort demonstrate strong negative correlations with fasting blood glucose, 1-hour postprandial, and 2-hour postprandial glucose levels, suggesting a co-regulatory mechanism. Unlike previous studies focusing on individual miRNA effects, our dual-miRNA approach reveals a synergistic interaction, providing mechanistic insights. This interaction between the molecular pathways of these miRNAs and their clinical manifestation, namely, the severity of maternal hyperglycemia, highlights an epigenetic connection between miRNA expression and glucose/lipid dysregulation. This broadens the understanding beyond conventional biochemical markers, offering new perspectives on GDM’s molecular complexity and identifications of potential therapeutic targets.

In addition to glucose and lipid metabolism pathways, the enrichment of target genes in biological processes related to nervous system development, such as synaptic membrane, neuron to neuron synapse, post-synaptic specialization, axon guidance and asymmetric synapse, is an unexpected but intriguing finding. This not only highlights the fundamental functions of the nervous system but also touches upon the complexity of neural activity. Abnormalities in these biological processes may lead to neurodevelopmental issues in the offspring, consistent with the potential neurobehavioral problems that GDM fetuses may encounter after birth. Our observation aligns with emerging evidence suggesting that GDM may have long-term neurodevelopmental consequences for offspring, leading to adverse outcomes such as learning disabilities, memory impairment, language development abnormalities, motor problems, and antisocial behavior [48]. Moreover, axon guidance is critically related to pancreatic islet cell morphology. Axon guidance molecules, such as Semaphorins, Ephrins, and netrins, play crucial roles in regulating the migration of endocrine progenitor cells, helping to establish the correct structure of pancreatic islet cells, and directly affecting the communication between mature islet cells, thereby regulating insulin secretion [49]. This point is preliminarily confirmed in our data. Furthermore, our analysis also revealed the enrichment of the Rap1 signaling pathway. Rap1 belongs to the small GTPase family, which a study reported to play an important role in regulating insulin secretion [50]. These findings provide a novel perspective on the complex interplay between neural and metabolic processes in GDM.

Although direct evidence of miR-326 and miR-532-3p dysregulation in placenta tissue or pancreatic islets in GDM is currently lacking in scientific literature or publicly accessible datasets, circulating miRNAs have consistently been shown to mirror placental miRNA profiles associated with pregnancy complications, including GDM [51]. The choice to utilize peripheral blood sampling, being a non-invasive approach, was intentional to enhance clinical utility and patient compliance, which are critical factors for a viable screening modality. In this context, the clinical and translational significance of our developed qPCR assay is underscored by its high diagnostic accuracy, combined with operational advantages, positioning it as a novel tool for GDM screening. Unlike the traditional OGTT, a molecular assay targeting miR-326 and miR-532-3p offers enhanced simplicity, rapidity, and patient adherence. Furthermore, the assay employs standard SYBR-Green qPCR chemistry and necessitates only two targets along with an endogenous reference gene, making it one of the most economical molecular diagnostic platforms currently available. Its cost-efficiency and reproducibility support feasible implementation across diverse healthcare settings, especially those with limited resources. The straightforward procedure, rapid processing time, reproducibility and affordability facilitate earlier and more accurate GDM diagnosis, thereby improving maternal and neonatal health outcomes. This assay holds promise as a complementary diagnostic approach alongside existing screening protocols. Future research should focus on integrating matched plasma and placental tissue or pancreatic islet models to definitively establish the relationship between circulating miRNA levels and tissue-specific regulation.

Although our case-control study provides compelling evidence, several limitations should be acknowledged. First and foremost is the absence of in vitro functional assays to mechanistically validate the link between miR-326, miR-532-3p, and GDM pathogenesis. While our expanded bioinformatics analysis (now using a robust consensus from TargetScan, miRDB, and DIANA-microT 2023) and the strong negative correlations with patient glycemic levels provide compelling evidence linking to the PI3K/Akt signaling pathway, this association requires direct experimental confirmation. Without this functional data, which is beyond our current infrastructural capacity, the mechanistic insights of our current study remain hypothesis-generating, as they do not account for post-transcriptional complexities or alternative pathways that could influence GDM pathophysiology. Despite this limitation beyond our current capacity, this foundational clinical investigation provides a strong mechanistic rationale and validated biomarkers essential for progressing this critical research pathway. We are actively designing subsequent studies to elucidate the specific molecular mechanism. This multi-step validation will initially utilize dual-luciferase reporter assays, likely employing a BeWo trophoblast cell line, to verify direct interactions between miR-326 and miR-532-3p with the 3’-untranslated regions (UTR) of key PI3K/Akt-related target genes. Following this, gain-of-function studies using miRNA mimics will be conducted to determine their direct effect on the total and phosphorylated protein levels of core pathway components, such as PI3K and Akt, through Western blot analysis. Finally, to confirm the specificity of these regulatory effects via target gene modulation, rescue experiments involving co-transfection of the miRNA mimics with siRNAs against the target genes will be performed to evaluate whether pathway activity can be restored.

Second, regarding generalizability, the single-centre cohort (*n* = 110), while statistically powered and rigorously matched for exploratory analysis, was recruited from a specific region in Southeast China (Putian city) and may not encompass the heterogeneity of GDM subtypes (e.g., insulin-sensitive versus resistant) or comorbidities (e.g., hypertension, thyroid disease). We recognize that genetic heterogeneity, diet patterns, and lifestyle factors vary across populations, influencing miRNA expression profiles. Although our findings were cross-validated with two independent GEO datasets (Fig. 2D and E) and mechanistically linked to the highly conserved PI3K/Akt pathway [52], the diagnostic thresholds established herein require validation in larger, multi-centre, ethnically diverse cohorts to improve transferability.

Furthermore, the cross-sectional design confirms the correlation between miRNA expression at 24–28 weeks of gestation, aligning with the OGTT window, but does not substantiate predictive capacity in early pregnancy, an area requiring future investigation. To address these limitations, we are actively planning a multi-centre, prospective longitudinal cohort study involving pregnant women in the first trimester (10–12 weeks) across multiple (> 5) tertiary hospitals in various Chinese regions. Applying the strict inclusion/exclusion criteria of the current study, we will collect baseline peripheral blood samples, quantify miR-326 and miR-532-3p Ct values, and monitor participants until the 24–28 week follow-up. GDM diagnosis will be definitively established using the OGTT as the gold standard, per the latest ADA 2024 guidelines [53], and analyzed for early predictive value through Ct values and AUCs analysis. This comprehensive prospective approach will be fundamental in validating the early predictive utility of this miRNA signature, potentially establishing it as a first-trimester screening tool.

## Conclusion

This study identifies and validates circulating miR-326 and miR-532-3p as a synergistic pair of biomarkers for GDM with high diagnostic accuracy through a cost-effective two-phase methodology. By linking these miRNAs to PI3K/Akt and Rap1 signaling pathways, we illuminate GDM’s metabolic-neural complexity, offering the first dual-miRNA perspective on its pathogenesis. Our developed qPCR assay promises a feasible, accessible complementary tool for GDM screening. Ultimately, these validated miRNAs represent a step toward improved GDM management, emphasizing early detection and facilitating targeted therapeutic interventions. Future investigations should prioritize external validation through multi-centre cohorts in the first trimester to determine population and subtype applicability, integrating these microRNA signatures with proteomic analyses to develop a comprehensive multi-omic model of GDM pathogenesis. This approach aims to link upstream epigenetic regulators with downstream functional protein networks, fostering early intervention strategies to improve maternal-fetal health outcomes.

## Supplementary Information

Below is the link to the electronic supplementary material.

Supplementary Material 1

## Data Availability

The manuscript encompasses all necessary data. Raw experimental data, including export files of RNA-seq, PCR reactions, and derived data supporting the findings of this study can be available from the corresponding authors H-Z. L and L. X upon reasonable request.
