# Supplementary material for: Circulating miR-326 and miR-532-3p: novel biomarkers for early gestational diabetes mellitus detection with high diagnostic accuracy
Source: J Endocrinol Invest. 2026 Feb 5;49(7):1635–50. doi: 10.1007/s40618-026-02829-z (PMC13451328; doi:10.1007/s40618-026-02829-z)
Supplement: Supplementary file 1 [file 40618_2026_2829_MOESM1_ESM.docx]

**Supplementary information**

**Additional file 1: Supplementary results**

**Figure S1: Bootstrap resampling evaluation of the diagnostic robustness of miR-326 and miR-532-3p.** (A) ROC curves for miR-326 generated from 1000 iterations of bootstrap resampling, based on qPCR Ct values, demonstrating high diagnostic stability. (B) Plot showing the minimal variability in ROC curves for miR-326 across the 1000 bootstrap iterations. (C) ROC curves for miR-532-3p generated from 1000 iterations of bootstrap resampling. (D) Plot showing the consistency of AUC curves for miR-532-3p across the 1000 bootstrap iterations.

**Figure S2: Venn diagram of consensus target gene prediction.** Intersection of putative target genes for (A) miR-326 and (B) miR-532-3p predicted by three independent databases: TargetScan, miRDB, and DIANA-microT 2023. A consensus-based approach was applied, identifying high-confidence target genes (central intersection) for downstream GO and KEGG enrichment analyses.

Supplementary Table 1: Comparison of pre-pregnancy and post-pregnancy weight and BMI within the GDM and Control groups.

| Group | Pre-pregnancy (mean ± SD) | Post-pregnancy (mean ± SD) | P value |
| --- | --- | --- | --- |
| GDM group weight (kg) | 57.06(8.10) | 57.76(9.04) | 0.040 |
| Control group weight (kg) | 54.95(8.94) | 56.16(9.05) | <0.001 |
| GDM group BMI (kg/m^2^) | 21.97(2.96) | 22.23(3.28) | 0.041 |
| Control group BMI (kg/m^2^) | 21.26(3.19) | 21.73(3.26) | <0.001 |

Supplementary Table 2: Complete list of 229 differentially expressed miRNAs identified by RNA-sequencing.

| miRNA | P value | FDR^*^ | Log_2_ FC^*^ | Regulated |
| --- | --- | --- | --- | --- |
| hsa-miR-5193 | 1.929473 | 0.005457 | 0.000129 | up |
| hsa-miR-5003-3p | 1.49168 | 0.056533 | 0.002596 | up |
| hsa-miR-3127-5p | 1.472351 | 0.071396 | 0.003701 | up |
| hsa-miR-5690 | 1.470262 | 0.028745 | 0.00098 | up |
| novel_miR_96 | 1.445824 | 0.071396 | 0.003655 | up |
| hsa-miR-6734-5p | 1.444353 | 0.001272 | 1.89E-05 | up |
| hsa-miR-122-5p | 1.427898 | 0.086312 | 0.004604 | up |
| hsa-miR-4755-3p | 1.364167 | 0.0901 | 0.005003 | up |
| hsa-miR-320a-3p | 1.346963 | 9.20E-07 | 3.62E-09 | up |
| novel_miR_562 | 1.340167 | 0.087916 | 0.004766 | up |
| hsa-miR-5009-5p | 1.338308 | 0.002045 | 3.31E-05 | up |
| novel_miR_89 | 1.296268 | 0.105363 | 0.006312 | up |
| novel_miR_274 | 1.276085 | 0.135335 | 0.009054 | up |
| novel_miR_358 | 1.222355 | 0.067548 | 0.003207 | up |
| hsa-miR-939-5p | 1.221384 | 9.72E-05 | 9.35E-07 | up |
| hsa-miR-4498 | 1.214836 | 0.208765 | 0.016339 | up |
| hsa-miR-3177-3p | 1.184024 | 0.017187 | 0.000514 | up |
| hsa-miR-6509-3p | 1.178338 | 0.231627 | 0.019591 | up |
| novel_miR_336 | 1.166489 | 0.231627 | 0.019648 | up |
| novel_miR_65 | 1.166489 | 0.231627 | 0.019648 | up |
| hsa-miR-6726-3p | 1.155045 | 0.136171 | 0.009169 | up |
| novel_miR_522 | 1.14844 | 0.242112 | 0.02118 | up |
| hsa-miR-2110 | 1.146324 | 1.46E-07 | 4.46E-10 | up |
| novel_miR_476 | 1.132844 | 0.272265 | 0.025821 | up |
| novel_miR_722 | 1.12257 | 0.267668 | 0.024344 | up |
| novel_miR_793 | 1.111905 | 0.232921 | 0.019918 | up |
| novel_miR_59 | 1.111462 | 0.097543 | 0.005715 | up |
| novel_miR_200 | 1.096043 | 0.231627 | 0.019458 | up |
| hsa-miR-6803-3p | 1.095318 | 0.165525 | 0.01187 | up |
| hsa-miR-320b | 1.095155 | 4.85E-05 | 3.78E-07 | up |
| hsa-miR-4685-3p | 1.091228 | 0.015102 | 0.000436 | up |
| novel_miR_315 | 1.087919 | 0.055315 | 0.002419 | up |
| novel_miR_705 | 1.081181 | 0.278613 | 0.027289 | up |
| hsa-miR-331-3p | 1.072456 | 3.90E-06 | 2.22E-08 | up |
| hsa-miR-6894-3p | 1.061704 | 0.294964 | 0.030309 | up |
| novel_miR_204 | 1.061246 | 0.270641 | 0.025088 | up |
| hsa-miR-3146 | 1.044722 | 0.338493 | 0.035978 | up |
| novel_miR_352 | 1.039116 | 0.272265 | 0.025838 | up |
| novel_miR_670 | 1.037739 | 0.278613 | 0.027167 | up |
| hsa-miR-1270 | 1.037732 | 0.131606 | 0.008689 | up |
| novel_miR_28 | 1.032361 | 0.291611 | 0.029569 | up |
| hsa-miR-4440 | 1.02972 | 0.356866 | 0.042825 | up |
| hsa-miR-1229-3p | 1.019551 | 0.165747 | 0.011958 | up |
| hsa-miR-5090 | 1.019227 | 0.259085 | 0.023337 | up |
| novel_miR_368 | 1.015481 | 0.356866 | 0.04334 | up |
| hsa-miR-5187-5p | 1.01536 | 0.356866 | 0.044472 | up |
| novel_miR_691 | 1.012544 | 0.356866 | 0.043779 | up |
| novel_miR_471 | 0.997691 | 0.306654 | 0.031778 | up |
| hsa-miR-3140-3p | 0.987205 | 0.038952 | 0.001465 | up |
| hsa-miR-299-3p | 0.979138 | 0.356866 | 0.044229 | up |
| hsa-miR-629-5p | 0.967243 | 0.000101 | 1.01E-06 | up |
| hsa-miR-3180 | 0.962199 | 0.338493 | 0.036262 | up |
| hsa-miR-3180-3p | 0.962199 | 0.338493 | 0.036262 | up |
| novel_miR_878 | 0.954244 | 0.272265 | 0.025953 | up |
| hsa-let-7b-3p | 0.953883 | 0.044778 | 0.001762 | up |
| novel_miR_795 | 0.953516 | 0.355046 | 0.038933 | up |
| novel_miR_594 | 0.951015 | 0.184129 | 0.013928 | up |
| hsa-miR-3127-3p | 0.938933 | 0.175586 | 0.012822 | up |
| novel_miR_137 | 0.933389 | 0.060077 | 0.002785 | up |
| novel_miR_702 | 0.932507 | 0.356866 | 0.040817 | up |
| hsa-miR-326 | 0.930544 | 0.023352 | 0.000756 | up |
| hsa-miR-15b-3p | 0.926719 | 4.85E-05 | 3.82E-07 | up |
| hsa-miR-4669 | 0.926644 | 0.37383 | 0.0481 | up |
| novel_miR_381 | 0.916058 | 0.005542 | 0.000133 | up |
| hsa-miR-423-5p | 0.915971 | 3.90E-06 | 2.10E-08 | up |
| novel_miR_500 | 0.914709 | 0.356866 | 0.044291 | up |
| novel_miR_180 | 0.914239 | 0.356866 | 0.041637 | up |
| hsa-miR-5010-5p | 0.908556 | 0.224289 | 0.018045 | up |
| hsa-miR-4664-3p | 0.905863 | 0.356866 | 0.043352 | up |
| hsa-miR-1343-3p | 0.899442 | 0.278613 | 0.027026 | up |
| hsa-miR-6855-5p | 0.898651 | 0.291409 | 0.029052 | up |
| hsa-miR-6842-5p | 0.889715 | 0.356866 | 0.043987 | up |
| hsa-miR-1224-5p | 0.887851 | 0.11097 | 0.006793 | up |
| hsa-miR-6799-3p | 0.885677 | 0.270641 | 0.025016 | up |
| hsa-miR-10401-3p | 0.871011 | 0.217809 | 0.017191 | up |
| novel_miR_287 | 0.861854 | 0.356866 | 0.040252 | up |
| hsa-miR-550a-3p | 0.861185 | 0.049122 | 0.002062 | up |
| hsa-miR-3615 | 0.845548 | 0.003886 | 8.16E-05 | up |
| hsa-miR-574-5p | 0.834058 | 0.011282 | 0.000306 | up |
| hsa-miR-877-5p | 0.824842 | 0.029521 | 0.00102 | up |
| hsa-miR-548at-5p | 0.822441 | 0.291409 | 0.02902 | up |
| novel_miR_786 | 0.82014 | 0.363212 | 0.046046 | up |
| hsa-let-7b-5p | 0.819462 | 0.002804 | 5.64E-05 | up |
| hsa-miR-16-2-3p | 0.807764 | 0.071396 | 0.003715 | up |
| hsa-miR-766-3p | 0.803975 | 0.11097 | 0.006788 | up |
| hsa-miR-6741-5p | 0.803571 | 0.356866 | 0.041852 | up |
| hsa-miR-1292-5p | 0.799775 | 0.001893 | 2.98E-05 | up |
| novel_miR_717 | 0.797595 | 0.243825 | 0.021643 | up |
| hsa-miR-1307-3p | 0.793847 | 0.0357 | 0.00128 | up |
| hsa-miR-6877-5p | 0.774674 | 0.190412 | 0.014487 | up |
| hsa-miR-184 | 0.763449 | 0.177393 | 0.013116 | up |
| hsa-miR-147b-3p | 0.751145 | 0.018723 | 0.000584 | up |
| hsa-miR-664a-5p | 0.742848 | 0.242727 | 0.021439 | up |
| hsa-miR-491-5p | 0.729068 | 0.276674 | 0.026494 | up |
| novel_miR_884 | 0.724656 | 0.154954 | 0.010841 | up |
| novel_miR_616 | 0.707898 | 0.115104 | 0.007247 | up |
| hsa-miR-486-5p | 0.705002 | 0.010166 | 0.000267 | up |
| novel_miR_617 | 0.700199 | 0.120394 | 0.007633 | up |
| hsa-let-7d-3p | 0.691029 | 0.070756 | 0.003434 | up |
| hsa-miR-3940-3p | 0.690943 | 0.056219 | 0.002507 | up |
| hsa-miR-636 | 0.686386 | 0.071396 | 0.003538 | up |
| hsa-miR-1285-5p | 0.681106 | 0.368898 | 0.047262 | up |
| hsa-miR-185-3p | 0.671823 | 0.025028 | 0.000821 | up |
| hsa-miR-6815-5p | 0.668146 | 0.355046 | 0.039251 | up |
| hsa-miR-532-3p | 0.657572 | 0.044778 | 0.001759 | up |
| hsa-miR-1273c | 0.635898 | 0.278613 | 0.027131 | up |
| hsa-miR-744-5p | 0.63526 | 0.008796 | 0.000223 | up |
| novel_miR_361 | 0.624199 | 0.294964 | 0.030295 | up |
| hsa-miR-4732-5p | 0.599644 | 0.050736 | 0.002196 | up |
| hsa-miR-652-3p | 0.599176 | 0.048579 | 0.001997 | up |
| novel_miR_119 | 0.598483 | 0.231627 | 0.019648 | up |
| novel_miR_375 | 0.598483 | 0.231627 | 0.019648 | up |
| hsa-miR-4746-5p | 0.593083 | 0.094958 | 0.005447 | up |
| novel_miR_355 | 0.586605 | 0.37383 | 0.048384 | up |
| hsa-miR-1271-5p | -0.60317 | 0.368898 | 0.047135 | down |
| hsa-miR-581 | -0.6091 | 0.067548 | 0.003249 | down |
| hsa-miR-28-5p | -0.62095 | 0.055635 | 0.002457 | down |
| hsa-miR-152-3p | -0.62653 | 0.351549 | 0.038122 | down |
| hsa-miR-651-5p | -0.62681 | 0.041418 | 0.001594 | down |
| hsa-miR-101-3p | -0.6289 | 0.012375 | 0.000341 | down |
| hsa-miR-24-2-5p | -0.62941 | 0.018464 | 0.000565 | down |
| hsa-miR-374a-3p | -0.63032 | 0.090257 | 0.005091 | down |
| hsa-miR-32-3p | -0.63827 | 0.294964 | 0.030264 | down |
| hsa-miR-98-5p | -0.63998 | 1.14E-05 | 7.46E-08 | down |
| hsa-miR-5001-3p | -0.64005 | 0.356866 | 0.04047 | down |
| hsa-miR-144-3p | -0.65078 | 0.050204 | 0.002151 | down |
| hsa-miR-424-5p | -0.65274 | 0.356866 | 0.044436 | down |
| hsa-miR-27b-3p | -0.65737 | 0.000771 | 1.08E-05 | down |
| hsa-miR-9903 | -0.66279 | 0.263605 | 0.023859 | down |
| novel_miR_700 | -0.66656 | 0.036934 | 0.00134 | down |
| hsa-miR-451a | -0.66996 | 0.045437 | 0.001848 | down |
| novel_miR_819 | -0.67108 | 0.034584 | 0.001225 | down |
| novel_miR_836 | -0.69007 | 0.011038 | 0.000294 | down |
| hsa-miR-18a-5p | -0.6942 | 0.007098 | 0.000177 | down |
| hsa-miR-1278 | -0.69762 | 0.004959 | 0.000108 | down |
| hsa-miR-181a-2-3p | -0.70181 | 0.002135 | 3.58E-05 | down |
| hsa-miR-29b-3p | -0.70402 | 0.002552 | 4.97E-05 | down |
| hsa-miR-190a-5p | -0.70521 | 0.05624 | 0.002533 | down |
| hsa-miR-3143 | -0.7076 | 0.129835 | 0.008459 | down |
| hsa-miR-339-5p | -0.71004 | 0.049624 | 0.002105 | down |
| hsa-miR-21-5p | -0.7201 | 0.000661 | 8.38E-06 | down |
| hsa-miR-618 | -0.72142 | 0.222939 | 0.017742 | down |
| hsa-miR-188-5p | -0.72162 | 0.356866 | 0.044443 | down |
| hsa-let-7f-5p | -0.72654 | 0.000252 | 2.75E-06 | down |
| hsa-miR-142-3p | -0.73069 | 0.010166 | 0.000267 | down |
| hsa-miR-4742-5p | -0.73976 | 0.360333 | 0.045246 | down |
| hsa-miR-18b-5p | -0.7561 | 0.094958 | 0.005481 | down |
| hsa-miR-1260b | -0.76117 | 0.142766 | 0.009801 | down |
| hsa-miR-126-3p | -0.77051 | 0.000726 | 9.84E-06 | down |
| hsa-miR-99a-5p | -0.77198 | 0.015293 | 0.000448 | down |
| hsa-miR-2277-5p | -0.78112 | 0.356866 | 0.040811 | down |
| hsa-miR-450a-5p | -0.79547 | 0.071396 | 0.003574 | down |
| hsa-miR-125a-5p | -0.80351 | 0.071396 | 0.003522 | down |
| hsa-miR-24-1-5p | -0.80851 | 0.094958 | 0.005468 | down |
| hsa-miR-548al | -0.81174 | 0.242112 | 0.021279 | down |
| hsa-miR-4677-5p | -0.82335 | 0.270641 | 0.02493 | down |
| hsa-miR-141-3p | -0.82626 | 0.356866 | 0.041446 | down |
| hsa-miR-660-5p | -0.85124 | 0.002214 | 3.97E-05 | down |
| hsa-miR-301a-3p | -0.85283 | 0.087916 | 0.004773 | down |
| hsa-miR-5695 | -0.8588 | 0.022865 | 0.00073 | down |
| hsa-miR-450b-5p | -0.85906 | 0.071396 | 0.003642 | down |
| novel_miR_219 | -0.86426 | 0.356866 | 0.041071 | down |
| hsa-miR-3909 | -0.86777 | 0.06528 | 0.003054 | down |
| hsa-miR-628-5p | -0.86785 | 0.111641 | 0.006905 | down |
| hsa-miR-32-5p | -0.8704 | 0.002135 | 3.64E-05 | down |
| novel_miR_47 | -0.87141 | 0.346163 | 0.037343 | down |
| hsa-miR-340-3p | -0.87595 | 5.22E-05 | 4.33E-07 | down |
| hsa-miR-181a-3p | -0.87634 | 0.073217 | 0.003842 | down |
| novel_miR_400 | -0.89046 | 0.356866 | 0.042823 | down |
| novel_miR_532 | -0.89064 | 0.014739 | 0.000419 | down |
| novel_miR_446 | -0.9002 | 0.356866 | 0.0404 | down |
| novel_miR_141 | -0.90253 | 0.291611 | 0.029582 | down |
| hsa-miR-548aq-3p | -0.90568 | 0.291611 | 0.029554 | down |
| hsa-miR-548n | -0.90794 | 0.346163 | 0.037386 | down |
| hsa-miR-486-3p | -0.92519 | 0.272265 | 0.025517 | down |
| novel_miR_233 | -0.9264 | 0.376145 | 0.048848 | down |
| hsa-miR-873-5p | -0.93596 | 0.363212 | 0.046151 | down |
| hsa-miR-335-5p | -0.93725 | 0.001218 | 1.76E-05 | down |
| novel_miR_668 | -0.94145 | 0.37383 | 0.048382 | down |
| hsa-miR-29a-3p | -0.95554 | 2.80E-05 | 1.96E-07 | down |
| hsa-miR-301a-5p | -0.95584 | 0.003888 | 8.33E-05 | down |
| hsa-miR-340-5p | -0.95999 | 1.66E-06 | 7.26E-09 | down |
| hsa-miR-499a-5p | -0.96704 | 0.005407 | 0.000125 | down |
| hsa-miR-20a-5p | -0.96947 | 3.08E-06 | 1.48E-08 | down |
| hsa-miR-126-5p | -0.97405 | 0.00049 | 6.00E-06 | down |
| hsa-miR-4433b-3p | -0.97934 | 0.071396 | 0.00369 | down |
| hsa-miR-514a-3p | -0.97963 | 0.098549 | 0.005817 | down |
| novel_miR_49 | -0.98512 | 0.355046 | 0.039277 | down |
| novel_miR_227 | -1.00391 | 0.337177 | 0.035531 | down |
| hsa-miR-335-3p | -1.00478 | 0.002552 | 5.02E-05 | down |
| novel_miR_298 | -1.00792 | 0.005384 | 0.000122 | down |
| novel_miR_556 | -1.00792 | 0.005384 | 0.000122 | down |
| novel_miR_425 | -1.00887 | 0.356866 | 0.039829 | down |
| hsa-miR-125b-5p | -1.03271 | 0.0901 | 0.004972 | down |
| novel_miR_519 | -1.03694 | 0.002946 | 6.05E-05 | down |
| hsa-miR-374a-5p | -1.05733 | 4.86E-07 | 1.70E-09 | down |
| hsa-miR-106a-3p | -1.06699 | 0.014324 | 0.000401 | down |
| novel_miR_604 | -1.06824 | 0.002531 | 4.76E-05 | down |
| hsa-miR-369-5p | -1.07983 | 0.321381 | 0.033586 | down |
| hsa-miR-598-3p | -1.0819 | 0.002377 | 4.37E-05 | down |
| hsa-miR-3684 | -1.08365 | 0.272265 | 0.025614 | down |
| novel_miR_828 | -1.08764 | 0.133563 | 0.008877 | down |
| hsa-miR-31-5p | -1.09044 | 0.228528 | 0.018486 | down |
| novel_miR_765 | -1.10417 | 0.278613 | 0.026824 | down |
| novel_miR_777 | -1.13765 | 0.154756 | 0.010759 | down |
| hsa-miR-146a-3p | -1.17388 | 0.14226 | 0.009704 | down |
| novel_miR_666 | -1.20316 | 0.156684 | 0.011099 | down |
| hsa-miR-136-3p | -1.24216 | 0.183518 | 0.013802 | down |
| hsa-miR-624-5p | -1.25432 | 0.000121 | 1.27E-06 | down |
| novel_miR_835 | -1.25549 | 0.177393 | 0.013153 | down |
| hsa-miR-590-3p | -1.27301 | 9.50E-05 | 8.72E-07 | down |
| hsa-miR-5100 | -1.29678 | 0.032256 | 0.001128 | down |
| hsa-miR-4443 | -1.30376 | 0.146833 | 0.010144 | down |
| hsa-miR-199b-5p | -1.30881 | 6.52E-05 | 5.70E-07 | down |
| novel_miR_391 | -1.3116 | 0.056368 | 0.002563 | down |
| novel_miR_29 | -1.34352 | 0.123046 | 0.007909 | down |
| hsa-miR-449a | -1.34641 | 0.111641 | 0.006932 | down |
| novel_miR_589 | -1.35318 | 0.040403 | 0.001537 | down |
| hsa-miR-27a-3p | -1.3675 | 4.03E-19 | 3.05E-22 | down |
| hsa-miR-627-3p | -1.52948 | 0.000286 | 3.25E-06 | down |
| novel_miR_830 | -1.57759 | 0.045437 | 0.00184 | down |
| novel_miR_249 | -1.62194 | 0.038309 | 0.001421 | down |
| novel_miR_311 | -1.66232 | 5.82E-06 | 3.57E-08 | down |
| hsa-miR-1246 | -1.67136 | 0.00043 | 5.07E-06 | down |
| novel_miR_340 | -1.74328 | 1.01E-09 | 1.76E-12 | down |
| hsa-miR-3613-5p | -1.91114 | 4.03E-19 | 3.52E-22 | down |
| novel_miR_480 | -2.03584 | 5.33E-08 | 1.40E-10 | down |
| hsa-miR-4454 | -2.46895 | 1.10E-08 | 2.41E-11 | down |
| hsa-miR-10395-3p | -2.95316 | 4.74E-15 | 6.22E-18 | down |

* FDR: False Discovery Rate; Log_2_ FC: Log_2_ Fold Change.
